# Chylous ascites as a rare complication of abdominal trauma in a 7‐year‐old girl: A case report

**DOI:** 10.1002/ccr3.7408

**Published:** 2023-07-02

**Authors:** Anahita Jafari, Hamid Reihani, Fereshteh Karbasian, Behnaz Darban, Seyed Mohsen Dehghani

**Affiliations:** ^1^ Gastroenterology and Hepatology Research Center Shiraz University of Medical Sciences Shiraz Iran; ^2^ School of Medicine Shiraz University of Medical Sciences Shiraz Iran; ^3^ Department of Pediatric Gastroenterology and Hepatology Iran University of Medical Sciences Tehran Iran; ^4^ Department of Pediatric Gastroenterology Hormozgan University of Medical Sciences Bandar Abbas Iran

**Keywords:** abdominal injuries, ascites, chylous ascites, pediatrics

## Abstract

**Key Clinical Message:**

Abdominal trauma can be one of the causes of chylous ascites in pediatric cases, along with tuberculosis and malignancy. However, a definitive diagnosis is more reasonable to be done by excluding other causes.

**Abstract:**

Chylous ascites (CA) is a rare type of ascites. Though it has high mortality and morbidity rates, which usually happen due to the rupture of lymph vessels into the peritoneal cavity. Congenital abnormalities, including lymphatic hypoplasia or dysplasia, are the most causes in pediatrics. CA following trauma in children is very rare, and to the best of our knowledge, there are very few reports in this regard. Here, we report a 7‐year‐old girl who was referred to our center with CA after a car accident.

## Graphical abstract

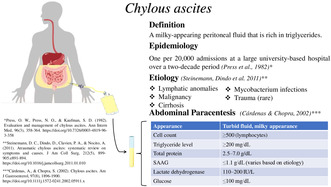

## 1 INTRODUCTION

Ascites is a common symptom of various disorders, but a chylous form is an uncommon condition, which usually happens due to the rupture of lymph vessels into the peritoneal cavity. Turbid milky ascites is the primary key for diagnosis.^1^ Chylous ascites (CA) is a rare type of ascites. But it has high mortality and morbidity rates. A study done by Press et al.^2^ reported that CA happens about 1 per 20,246 in general hospital admission. Also, this phenomenon is expected to grow due to an uprising trend in thoracic surgery.^3^, ^4^ In the 17th century most physicians believed that trauma was the leading cause of CA.^4^ CA has different etiologies. Malignancy, cirrhosis, tuberculosis, or trauma are the most critical causes in adults.^5^ Congenital abnormalities, including lymphatic hypoplasia or dysplasia, are the most causes in children.^6^ CA was divided into two groups: traumatic and atraumatic. Evaluation and long‐term management of CA are related to underlying etiology.^5^ Here, we report a 7‐year‐old girl who was referred to our center with CA after a car accident.

## 2 CASE PRESENTATION

A 7‐year‐old girl presented to the physician with bright red blood after defecating for 26 days beforehand. Her colonoscopy revealed erythematous mucosa and a few erosions on her rectum. In addition, biopsies from that site revealed nodular lymphoid hyperplasia and apoptotic colonopathy. She received some stool softeners and a topical agent. About 10 days later, the patient was involved in a car accident. Therefore, a computed tomography (CT) scan of the abdomen was performed due to abdominal trauma and revealed a massive amount of fluid in the abdominal cavity. The patient was discharged from the hospital after 3 days.

About 14 days later, the patient presented to the pediatric department with increasing abdominal distention.

On physical exam, the patient's blood pressure was 100/75 mmHg, respiratory rate 22, pulses rate 110 beats per minute, weight was 18 kg, and height was 111 cm, so her body mass index (BMI) was 14/6 kg/m^2^. On abdominal examination, she has moderate distension. Other parts of the physical examinations were normal. His blood test showed hemoglobin of 13.7 g per deciliter, white blood cells of 5000 cells/mm^3^, 55.1% neutrophils, and 37.4% lymphocytes. In addition, biochemical analyses were performed, which showed triglycerides 59 mg/dL, cholesterol 125 mg/dL, albumin 4.6 gr/L, amylase 110 u/L, lipase 25 u/L, LDH 479 u/L. The renal function test and urine analysis were all normal. The rest of the laboratory data are summarized in Table 1.

**TABLE 1:** The results of the patient's laboratory test.

	Laboratory results	
Laboratory tests	On admission	Normal value (SI) units
White blood cell [10^3^/uL]	5	4–10 × 10^9^
Red blood cell [10^6^/uL]	4.30	4.2–5.2 × 10^9^ female
Hemoglobin [g/dL]	13.7	12–16 g/dL female
Platelets [10^3^/uL]	257	150–450 × 10^9^
Total bilirubin [mg/dL]	0.6	0.1–1.2
Direct bilirubin [mg/dL]	0.4	<0.3
Alkaline phosphate [U/L]	363	180–1200 pediatric
Aspartate aminotransferase [U/L]	36	<31 female
Alanine aminotransferase [U/L]	18	<31 female
Total protein [g/dL]	7	6.4–8.3
Albumin [g/dL]	4.6	3.5–5.2
Amylase [U/L]	110	25–125
Lipase [U/L]	25	<60
Sodium [meq/L]	141	136–145
Potassium [meq/L]	4.9	3.5–5.5
Magnesium [mg/dL]	2.2	1.6–2.5
Lactate dehydrogenase [U/L]	479	125–220
Triglyceride [mg/dL]	59	<150 normal
Cholesterol [mg/dL]	125	150–200 normal
C‐reactive protein [mg/L]	1	<6

**FIGURE 1:**
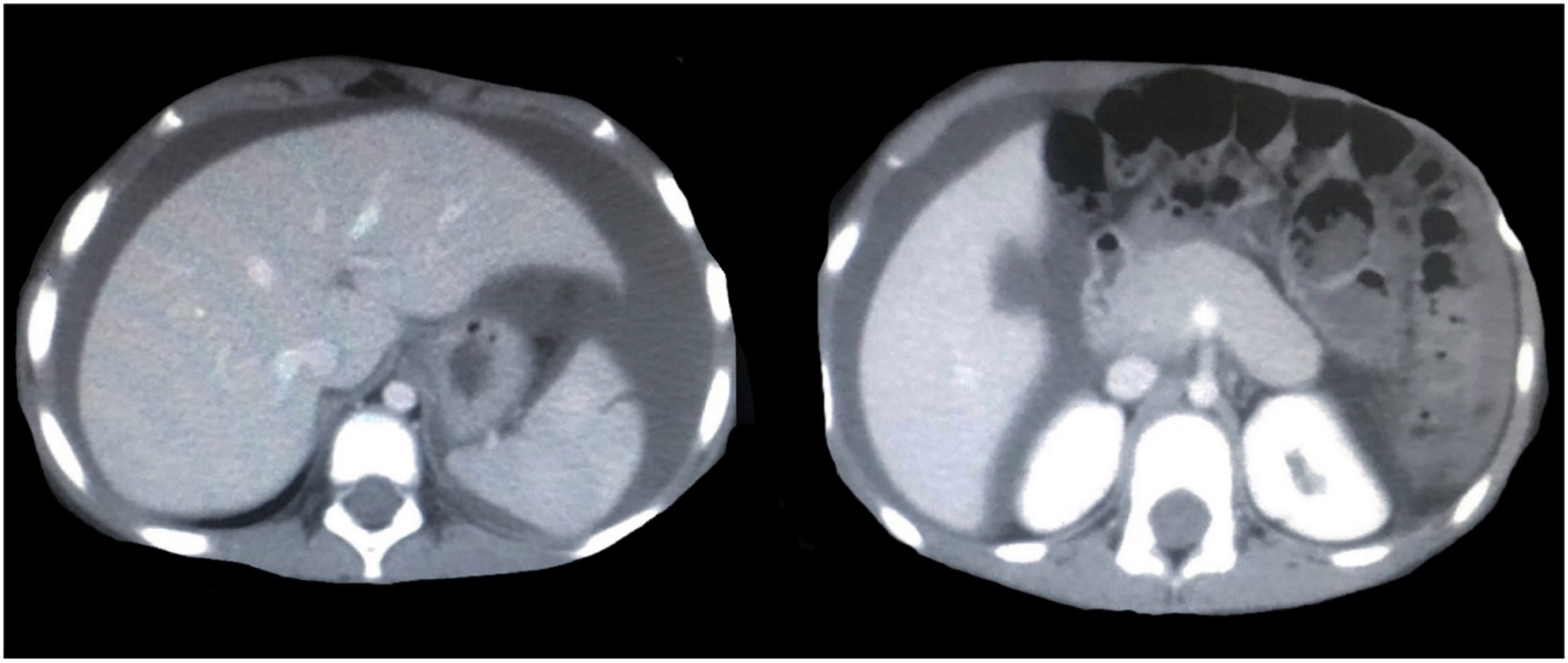
CT abdominopelvic report: evidence of severe free fluid is seen in the abdominopelvic cavity.

An abdominal ultrasound examination revealed moderate free fluid in the abdomen, so abdominal fluid was tapped under ultrasound guidance, and its laboratory parameters are presented in Table 2. An abdominal and pelvic computed tomography (CT) showed extensive free fluid in the abdominopelvic cavity (Figure 1).

**TABLE 2:** Characteristics of abdominal tap.

Appearance	Turbid fluid, milky appearance	
Abdominal fluid analysis		
Abdominal fluid count	Total cell count	16,750/mm^3^
	WBC count	925/mm^3^
		85% segment
		15% lymphocyte
	RBC count	15,825/mm^3^
Biochemistry	Fluid glucose	174 mg/dL
	Fluid LDH	318 IU/L
	Fluid albumin	4.4 gr/L
	Fluid TG	3600 mg
	Fluid cholesterol	95 mg
	Fluid amylase	78 U/L
Adenosine deaminase (ADA)		Negative

Cytology from the fluid showed some isolated and loose clusters of mesothelial cells, a few histiocytes with numerous lymphoblasts, and immature lymphocytes in favor of lymphoproliferative lesion, and the authorized pathologist recommended bone marrow aspiration. An aspiration of her bone marrow was done after suspicion of malignancy was raised. The marrow showed mild hypocellularity. According to flow cytometry, 2–3% of myeloid cells were immature.

Several factors can cause a CA. In our case, the three primary differential diagnoses were tuberculosis, malignancy, and trauma, so we launched different tests to determine our diagnosis and treat our patient. A tuberculosis smear, culture, and adenosine deaminase test (ADA) were performed to exclude tuberculosis. To establish the diagnosis of lymphoproliferative disorder from the fluid, bone marrow aspiration and flow cytometry were performed, which were not indicative of malignancy. As a result, the malignancy cause of CA was excluded, and the patient was treated with a diagnosis of traumatic CA and received medium‐chain triglyceride (MCT) oil and octreotide. After all, in the follow‐up 2 weeks later, abdominal ultrasonography reported mild free fluid. Ultimately, with the treatment continued for a month, there was no trace of free fluid in the abdominal cavity.

## 3 DISCUSSION

**FIGURE 2:**
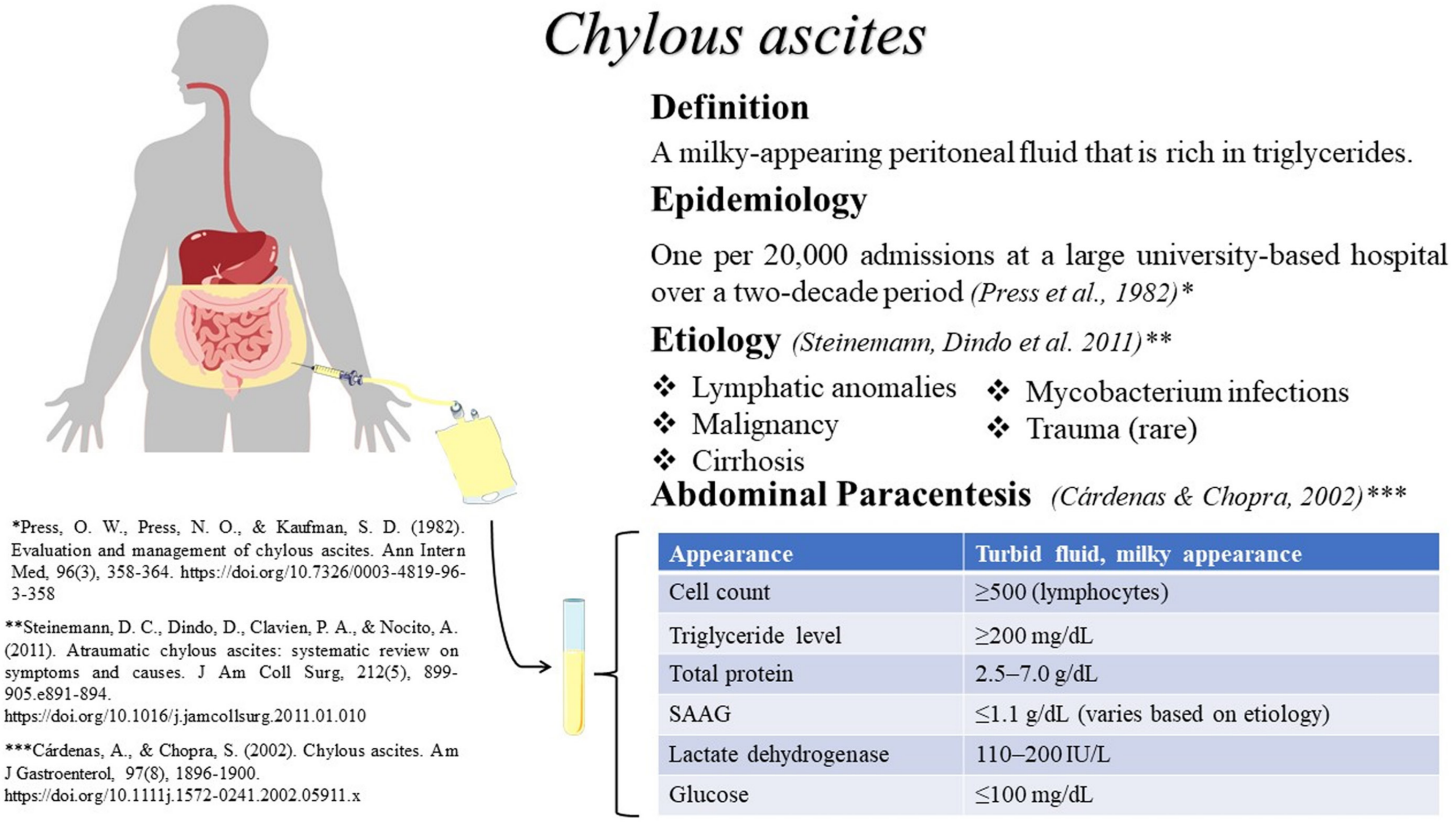
Characteristics of chylous ascites.^2^, ^8^, ^9^

As we know, CA is a rare type of ascites.^3^ So should be distinguished to find underlying causes. Cytology, cell count, Gram stain, culture, total protein concentration, albumin, glucose, LDH, triglyceride, and amylase should be checked in an ascetic fluid.^7^ But the most crucial laboratory test that can be used to confirm the diagnosis of CA is measuring the triglyceride level of fluid that be paracentesis from ascites. Most studies have cutoff >200 mg/dL.^1^ In our case, when we tapped her abdominal fluid, we saw turbid red fluid, and also, its analysis showed that ascetic fluid triglyceride levels were 3600 mg/dL, so it confirmed that our patient had CA. CA characteristics are illustrated as a graphical abstract in Figure 2.

For CA diagnosis, like the other diseases, history and physical exam should be taken carefully.^8^ In CA most common symptoms are abdominal distention and inspected abdominal pain.^5^ In children, abdominal distention is most common in atraumatic CA.^9^ Our patient presented with abdominal distention as a usual symptom of CA. The most common cause of CA in adults differs in Western and Eastern countries. Malignancy is at the top of diagnosis of CA in Western but infectious diseases like tuberculosis and filariasis are the most common causes in Eastern countries. In the children population, congenital abnormalities and trauma are the most critical etiologies for CA formation.^8^ Therefore, we considered all the possible etiologies and did additional workups.

Traumatic and atraumatic are two important etiology groups.^5^ In traumatic cases, postoperative and blunt trauma is the most critical etiology factor for CA. Surgery can lead to CA due to rupture or compression of lymphatic vessels.^8^ Blunt trauma is one of the common causes in children.^10^ CA due to trauma is highly uncommon.^11^ Although trauma is a rare cause, in this case, our patient had a car accident history, so we should consider this differential diagnosis. Abdominal or thoracic surgery can cause acute onset of CA.^12^ The rupturing mechanism in blunt trauma is hyperextension and hyperflexion of lymph vessels, but in surgery, direct injury to these vessels causes CA.^13^

One of the vital causes of CA is malignancy. Different solid malignancies, including lymphoma, neuroendocrine tumors, and sarcoma, can lead to CA, but among them, lymphoma has the highest prevalence, about one‐third of the cases.^1^ As we know, malignancy is a vital etiology, so we should not miss it. In this case, we are suspicious of it. So, we sent cytology from ascites fluid. And we did additional workups to exclude it.

Treatment of CA includes three parts: conservative, interventional, and surgical treatment.

Choosing a treatment method is to depend on the etiology of CA. Total parenteral nutrition (TPN), MCT diet, and octreotide can be used in conservative treatment. The surgical approach should be made in refractory CA.^10^

## 4 CONCLUSION

We still recommend that tuberculosis and malignancy are among the more important causes of CA. However, in rare cases like ours, trauma can be included in the differential diagnoses, and relevant work‐ups can be performed.

## AUTHOR CONTRIBUTIONS

**Anahita Jafari:** Conceptualization; writing – original draft; writing – review and editing. **Hamid Reihani:** Conceptualization; writing – original draft; writing – review and editing. **fereshteh karbasian:** Conceptualization; project administration; supervision; writing – original draft; writing – review and editing. **Behnaz Darban:** Conceptualization; writing – original draft; writing – review and editing. **Seyed Mohsen Dehghani:** Supervision; writing – original draft; writing – review and editing.

## FUNDING INFORMATION

None.

## CONFLICT OF INTEREST STATEMENT

The authors declare that they have no competing interests.

## ETHICS APPROVAL AND CONSENT TO PARTICIPATE

Our study has been reviewed and approved by the Medical Ethics Committee of Shiraz University of Medical Sciences.

## CONSENT FOR PUBLICATION

Written informed consent was obtained from the patient's parents to publish this Case report. A copy of the written consent is available for review and can be requested at any time by the journal's editor.

## Data Availability

Data of the patient can be requested from the authors. Do not hesitate to contact the corresponding author if you are interested in such data.
